# Pulse-shape discrimination against low-energy Ar-39 beta decays in liquid argon with 4.5 tonne-years of DEAP-3600 data

**DOI:** 10.1140/epjc/s10052-021-09514-w

**Published:** 2021-09-16

**Authors:** P. Adhikari, R. Ajaj, M. Alpízar-Venegas, P.-A. Amaudruz, D. J. Auty, M. Batygov, B. Beltran, H. Benmansour, C. E. Bina, J. Bonatt, W. Bonivento, M. G. Boulay, B. Broerman, J. F. Bueno, P. M. Burghardt, A. Butcher, M. Cadeddu, B. Cai, M. Cárdenas-Montes, S. Cavuoti, M. Chen, Y. Chen, B. T. Cleveland, J. M. Corning, D. Cranshaw, S. Daugherty, P. DelGobbo, K. Dering, J. DiGioseffo, P. Di Stefano, L. Doria, F. A. Duncan, M. Dunford, E. Ellingwood, A. Erlandson, S. S. Farahani, N. Fatemighomi, G. Fiorillo, S. Florian, T. Flower, R. J. Ford, R. Gagnon, D. Gallacher, P. García Abia, S. Garg, P. Giampa, D. Goeldi, V. Golovko, P. Gorel, K. Graham, D. R. Grant, A. Grobov, A. L. Hallin, M. Hamstra, P. J. Harvey, C. Hearns, T. Hugues, A. Ilyasov, A. Joy, B. Jigmeddorj, C. J. Jillings, O. Kamaev, G. Kaur, A. Kemp, I. Kochanek, M. Kuźniak, M. Lai, S. Langrock, B. Lehnert, A. Leonhardt, N. Levashko, X. Li, J. Lidgard, T. Lindner, M. Lissia, J. Lock, G. Longo, I. Machulin, A. B. McDonald, T. McElroy, T. McGinn, J. B. McLaughlin, R. Mehdiyev, C. Mielnichuk, J. Monroe, P. Nadeau, C. Nantais, C. Ng, A. J. Noble, E. O’Dwyer, G. Oliviéro, C. Ouellet, S. Pal, P. Pasuthip, S. J. M. Peeters, M. Perry, V. Pesudo, E. Picciau, M.-C. Piro, T. R. Pollmann, E. T. Rand, C. Rethmeier, F. Retière, I. Rodríguez-García, L. Roszkowski, J. B. Ruhland, E. Sánchez-García, R. Santorelli, D. Sinclair, P. Skensved, B. Smith, N. J. T. Smith, T. Sonley, J. Soukup, R. Stainforth, C. Stone, V. Strickland, M. Stringer, B. Sur, J. Tang, E. Vázquez-Jáuregui, S. Viel, J. Walding, M. Waqar, M. Ward, S. Westerdale, J. Willis, A. Zuñiga-Reyes, DEAP Collaboration

**Affiliations:** 1 Department of Physics, University of Alberta, Edmonton, AB T6G 2R3 Canada; 2 AstroCeNT, Nicolaus Copernicus Astronomical Center, Polish Academy of Sciences, Rektorska 4, 00-614 Warsaw, Poland; 3 Canadian Nuclear Laboratories Ltd, Chalk River, ON K0J 1J0 Canada; 4 Centro de Investigaciones Energéticas, Medioambientales y Tecnológicas, 28040 Madrid, Spain; 5 Department of Physics, Carleton University, Ottawa, ON K1S 5B6 Canada; 6 Physics Department, Università degli Studi “Federico II” di Napoli, 80126 Naples, Italy; 7 Physics Department, Università degli Studi di Cagliari, 09042 Cagliari, Italy; 8 INFN Laboratori Nazionali del Gran Sasso, 67100 Assergi, AQ Italy; 9 Department of Physics and Astronomy, Laurentian University, Sudbury, ON P3E 2C6 Canada; 10 Instituto de Física, Universidad Nacional Autónoma de México, A. P. 20-364, 01000 Mexico, D.F. Mexico; 11 National Research Centre Kurchatov Institute, Moscow, 123182 Russia; 12 National Research Nuclear University MEPhI, Moscow, 115409 Russia; 13 INFN Napoli, 80126 Naples, Italy; 14 INFN Cagliari, Cagliari, 09042 Italy; 15 PRISMA+ Cluster of Excellence and Institut für Kernphysik, Johannes Gutenberg-Universität Mainz, 55128 Mainz, Germany; 16 Physics Department, Princeton University, Princeton, NJ 08544 USA; 17 Department of Physics, Engineering Physics and Astronomy, Queen’s University, Kingston, ON K7L 3N6 Canada; 18 Royal Holloway University London, Egham Hill, Egham, Surrey TW20 0EX UK; 19 SNOLAB, Lively, ON P3Y 1M3 Canada; 20 University of Sussex, Sussex House, Brighton, East Sussex BN1 9RH UK; 21 TRIUMF, Vancouver, BC V6T 2A3 Canada; 22 Department of Physics, Technische Universität München, 80333 Munich, Germany; 23 Arthur B. McDonald Canadian Astroparticle Physics Research Institute, Queen’s University, Kingston, ON K7L 3N6 Canada; 24 BP2, National Centre for Nuclear Research, ul. Pasteura 7, 02-093 Warsaw, Poland; 25 INAF-Astronomical Observatory of Capodimonte, Salita Moiariello 16, 80131 Naples, Italy; 26 Present Address: Nuclear Science Division, Lawrence Berkeley National Laboratory, Berkeley, CA 94720 USA; 27 Present Address: Nikhef and the University of Amsterdam, Science Park, 1098 XG Amsterdam, The Netherlands; 28 SNOLAB, Lively, ON P3Y 1M3 Canada

## Abstract

The DEAP-3600 detector searches for the scintillation signal from dark matter particles scattering on a 3.3 tonne liquid argon target. The largest background comes from $^{39}\text{ Ar }$ beta decays and is suppressed using pulse-shape discrimination (PSD). We use two types of PSD estimator: the prompt-fraction, which considers the fraction of the scintillation signal in a narrow and a wide time window around the event peak, and the log-likelihood-ratio, which compares the observed photon arrival times to a signal and a background model. We furthermore use two algorithms to determine the number of photons detected at a given time: (1) simply dividing the charge of each PMT pulse by the mean single-photoelectron charge, and (2) a likelihood analysis that considers the probability to detect a certain number of photons at a given time, based on a model for the scintillation pulse shape and for afterpulsing in the light detectors. The prompt-fraction performs approximately as well as the log-likelihood-ratio PSD algorithm if the photon detection times are not biased by detector effects. We explain this result using a model for the information carried by scintillation photons as a function of the time when they are detected.

## Introduction

Liquid argon (LAr) is used as target material in several current and planned experiments related to the direct search for WIMP dark matter and studies of neutrino properties [1–10]. Argon provides excellent pulse shape discrimination (PSD) power, which allows separation of backgrounds in the form of electron recoils^1^ (ER) from the WIMP scattering signal expected to manifest as a nuclear-recoil (NR) event [1, 3, 5, 8, 11].

PSD in LAr is based on the large difference in lifetimes between the argon excimer’s singlet (approximately 6 ns) and triplet (approximately 1400–1600 ns in the literature) states. Due to this difference, singlet and triplet photons are well-separated in time and individual events’ singlet-to-triplet ratios can be estimated to high precision. The expectation value for the singlet-to-triplet ratio depends on the linear energy transfer (LET) of the exciting particle [12]. Thus, any estimator sensitive to an event’s singlet-to-triplet ratio will group events with different LET into distinct populations, allowing discrimination.

The dominant background in LAr-based dark matter detectors is the beta-decay of $^{39}\text{ Ar }$, at a rate of approximately 1 Bq/kg in atmospheric argon [13–15]. For a 3 tonne-year exposure, this amounts to $\mathcal {O}(10^8)$ events per keV in the region of interest (approximately 16–33 keV electron-equivalent energy) for dark matter search. PSD is well-suited to suppress this background, as the dark matter-induced NR events have a different LET and thus different singlet-to-triplet ratio than the beta-induced ER events.

Two types of methods are commonly used for PSD in liquid scintillators whose pulse shape is dominated by two exponential decay times: the fraction of light collected in a prompt or a late window compared to the total amount collected, and a likelihood analysis where the photon detection times are compared to a signal and a background template. The implementation of both methods in the context of LAr scintillation detectors with $\mathcal {O}(10$ kg) target mass has been discussed in [16, 17], and in [18] we derived a first-principles model for the statistical distribution of the prompt-fraction PSD parameter. Here, we show the performance of both methods using 840 livedays of data from the 3.3 tonne target mass DEAP-3600 detector (providing approximately $1.8\times 10^{8}$ $^{39}\text{ Ar }$ events per keV in the energy ROI after data selection cuts).

## The DEAP-3600 detector

We give a brief overview of the DEAP-3600 detector in this section and refer the reader to [11] for a more detailed description.

The DEAP-3600 detector is located 2 km underground at SNOLAB in Sudbury, Canada. The centre of the detector is a spherical volume 170 cm in diameter, which contains 3.3 tonnes of LAr. The scintillation light created in the LAr travels through the argon volume until it reaches the surface of the acrylic containment vessel. The inside acrylic surface is coated with TPB, which shifts the 128 nm scintillation light to the blue spectral region. The wavelength-shifted light is transmitted to 255 Hamamatsu R5912 high quantum-efficiency PMTs through a total of 50 cm of acrylic comprising the wall of the acrylic vessel and the acrylic light guides. The acrylic contains a UV-absorbing additive to suppress Cherenkov light produced in the vessel and light guides.

The inner detector is sealed inside a stainless steel sphere and suspended in an 8 m diameter water shielding tank. The tank serves as active muon veto and as passive neutron moderator.

The signals from the 255 PMTs are passed to custom amplifiers which stretch and amplify them, before digitization by CAEN V1720 digitizers at 4 ns resolution; enough to resolve single-photoelectron (SPE) pulses. Each PMT channel has a threshold of approximately 0.1 photoelectrons (PE). To trigger the detector, a charge approximately equal to at least 19 PE must be detected across all PMTs in a 177 ns sliding window. Upon triggering, data from all PMTs are digitized from $-$ 2.6 to + 14.4 $\upmu \hbox {s}$ relative to the trigger time. The digitized traces are written to disk for offline analysis.

## Data selection and analysis

### Data selection

The data used in this work was taken between November 2016 and March 2020 for an exposure of 4.5 tonne-years after event position cuts. The vast majority of recorded events are from $^{39}\text{ Ar }$ $\beta$-decays, with a small contribution of $\gamma$’s from radioactivity in detector materials [19]. The dataset contains both blinded and unblinded data. In the blinded part of the dataset, which makes up approximately 50% of the total, events within and near the dark matter signal region were removed from analysis. We nevertheless use this data because the blinding removes a negligible amount of $^{39}\text{ Ar }$ events. The effect of the blinding will be discussed in more detail in Sect. 5.4.

Events are selected using the following data cleaning cuts:

1. Low level DAQ cuts: the event must not overlap with any DAQ self-diagnostic triggers, data describing the digtitized event waveform must be complete, pulse finding algorithm must have succeeded;
2. Timing: event peak time (that is the peak of the scintillation time distribution) must be near the DAQ trigger time;
3. Position: the reconstructed event position must be within the LAr volume and at least 13 cm away from the inner detector surface;
4. Pile-up: at least 20 $\upmu \hbox {s}$ passed since the previous trigger, no more than three photons were detected in a time window from $-$ 2.6 to $-$ 1.0 $\upmu \hbox {s}$ before the event peak, and no more than one event peak is recorded within the digitization time window.

These cuts do not remove all non-ER backgrounds from the Dark-Matter signal region. For example, a population of alpha decays with degraded energy is still expected [10].

### Photon counting

We use two methods to count the scintillation photons in a PMT pulse. A standard pulse-finding algorithm yields the peak time and the area under the peak for each pulse from the PMTs [20]. A robust but naive way to count the number of photons in such a pulse is to divide the area under the peak, *Q*, by the mean of the SPE charge distribution, $Q_\text {SPE}$. We denote the number of PE obtained from charge division as $\text {q}_\text {PE}$.

1 $$\begin{aligned} \text {q}_\text {PE}= \frac{Q}{Q_\text {SPE}} \end{aligned}$$

$\text {q}_\text {PE}$ is a biased estimator for the number of scintillation photons. The bias is caused mainly by correlated noise in the PMT, in the form of afterpulsing (AP). The DEAP-3600 PMTs have an AP probability of approximately 10 % [11, 21]. Since AP occurs in specific time windows between 100 ns and 10,000 ns, it modifies the pulse-shape [22], and since AP is a statistical process, it also contributes to the variance of the $\text {q}_\text {PE}$ count. The bulk of the variance in $\text {q}_\text {PE}$, however, is due to the width of the SPE charge distribution. For DEAP-3600 PMTs this width is approximately $\sigma _\text {SPE}/Q_\text {SPE} \approx 0.43$.

A less biased estimate of the number of scintillation photons can be obtained using a likelihood analysis. The method is described in [17], and the implementation in DEAP-3600 is explained in detail in [20, 23, 24], and briefly summarized here. We assume that the number of PE in a pulse ($n_{\text {PE}}$) is composed of PE from scintillation photons ($n_{\text {Sc}}$) and signals from AP ($n_{\text {AP}}$). The time response of the wavelength shifter and PMT dark noise are minor effects here and not considered. Using Bayes’ theorem, we calculate how likely it is to observe $n_{\text {PE}}$ at the time of each pulse, given the pulse charge, the LAr scintillation probability density function (PDF), the times of preceding pulses, the AP time and charge PDF, and the SPE charge distribution of the PMT. For a pulse observed at a given time in the waveform, the posterior probability for $n_{\text {PE}}$ is

2 $$\begin{aligned} P(n_{\text {Sc}}+ n_{\text {AP}}=n_{\text {PE}}|Q) = \frac{p(Q|n_{\text {PE}})p(n_{\text {Sc}})p(n_{\text {AP}})}{p(Q)}\;. \end{aligned}$$

The values for $n_{\text {Sc}}$ and $n_{\text {AP}}$ are determined using the minimum mean square error estimator [24]. The $n_{\text {Sc}}$ values are real rather than natural numbers in this approach. The method differs slightly from what is described in [17, 23], where $n_{\text {Sc}}$ and $n_{\text {AP}}$ are estimated using the maximum a posteriori (MAP) estimator. In the MAP approach, there are regions in the pulse shape where a pulse is always more likely to originate from AP, and in these time regions, the algorithm will remove all scintillation photons.

### Energy and position reconstruction

The event window in DEAP-3600 is defined between -28 ns to +10,000 ns relative to the time of the event peak T$_0$. We denote the total number of PE in the event window as $N_{\text {PE}}$, or if referring to a particular PE counting method, as $N_{\text {qpe}}$ or $N_{\text {nsc}}$. T$_0$ and the event position in the detector are determined respectively based on the photon detection times across the PMT array and on the charge pattern of detected photons [10].

$N_{\text {PE}}$ is related to the energy deposited in the detector through the light yield. For events in the energy region of interest (ROI) for dark matter search, the light yield is approximately 6.05$n_{\text {Sc}}$/$\hbox {keV}_{\mathrm{ee}}$. All event energies are given in electron equivalent energy, denoted by $\hbox {keV}_{\mathrm{ee}}$.

We will use $N_{\text {nsc}}$ as the energy estimator throughout this report.

### Simulated WIMP recoils

A sample of WIMP recoil events is obtained through a Monte Carlo simulation (MC) of $^{40}\text{ Ar }$ recoils distributed uniformly in the LAr with a flat energy spectrum. The simulation accounts for the full response of the detector including scintillation, photon scattering, wavelength shifting, PMT and DAQ instrumental effects. The recoil quenching factor and the singlet/triplet ratios as a function of energy are taken from the SCENE measurements [25] at zero electric field. Validation of the WIMP simulation is described in [10], and systematic uncertainties due to mis-modelling of $^{40}\text{ Ar }$ recoil events are discussed in Sect. 5.4. The simulated data is analyzed with the same analysis flow as the real data.

## PSD parameter definitions

The different PSD methods each define algorithms for calculating a PSD Parameter (PP) based on the detection times of scintillation photons for each event. PSD power is based on the fact that different types of interactions produce distinguishable photon detection PDFs. We denote these PDFs $p(t)_{\text {nr}}$ and $p(t)_{\text {er}}$ for nuclear recoils and electron recoils respectively. A detailed discussion of $p(t)_{\text {er}}$ can be found in [22]. The singlet fraction, and therefore $p(t)_{\text {nr}}$ and $p(t)_{\text {er}}$, has a dependence on energy. For the construction of the prompt-fraction discriminator described in Sect. 4.1, the energy dependence is of minor importance, and just the average shapes of $p(t)_{\text {nr}}$ and $p(t)_{\text {er}}$ across the energy ROI are used. The log-likelihood analysis described in Sect. 4.2 is sensitive to the energy dependence. Hence the construction of $p(t)_{\text {nr}}$ and $p(t)_{\text {er}}$, including the energy-dependence, will be described there.

### Prompt fraction

The prompt fraction PP is commonly used in LAr-based detectors and is defined as

3 $$\begin{aligned} F_{\text {prompt}}= \frac{\sum _{t>t_\text {start}}^{t<t_\text {prompt}} n(t)}{\sum _{t>t_\text {start}}^{t<t_\text {total}} n(t)} \end{aligned}$$

where *n* can be either $n=\text {q}_\text {PE}{}$ or $n=n_{\text {Sc}}$, and all times are relative to the event peak time T$_0$.

The time $t_\text {prompt}$ is chosen such that the difference between the cumulative distribution functions of $p(t)_{\text {nr}}$ and $p(t)_{\text {er}}$, normalized to the standard deviation of the ER event distribution under a given time window,

4 $$\begin{aligned} \Delta (t_\text {prompt}) = \frac{1}{\sigma _\text {er}(t_\text {prompt}) }\int _{t_\text {start}}^{t_\text {prompt}} (p(t)_{\text {nr}}- p(t)_{\text {er}}) dt\;, \end{aligned}$$

is maximised.^2^ For LAr pulse shapes, this is the case for $t_\text {prompt}$ in the range of 60–80 ns.^3^

In DEAP-3600 $t_\text {start}=-28$ ns, the prompt window goes up to $t_\text {prompt}$=60 ns, and the full event window ends at $t_\text {total}$=10,000 ns. The denominator of Eq. (3) is thus equal to $N_{\text {PE}}$. $t_\text {start}$ is negative so that photons that are detected before the event time due to the finite timing resolution of the detector are also counted.

This method gives us two PPs:

- $$F_{\text {prompt}}$$ based on $$\text {q}_\text {PE}$$ ($$F^{\text {qpe}}_{\text {prompt}}$$)
- $$F_{\text {prompt}}$$ based on $$n_{\text {Sc}}$$($$F^{\text {nsc}}_{\text {prompt}}$$)

$F^{\text {qpe}}_{\text {prompt}}$with a prompt time window of 150 ns was used in [18] and in [26], while [10] used $F^{\text {nsc}}_{\text {prompt}}$ with a 60 ns prompt window and [27] used $F^{\text {qpe}}_{\text {prompt}}$with a 90 ns prompt window.

### Log-likelihood-ratio

The construction of the log-likelihood-ratio $L_{\text {recoil}}$ PP is described in [17, 24]. Namely

5 $$\begin{aligned} L_{\text {recoil}}= \frac{1}{2} \cdot \bigg (1 + \frac{\sum _{t>t_\text {start}}^{t<t_\text {total}} w(t) n(t)}{\sum _{t>t_\text {start}}^{t<t_\text {total}} n(t)}\bigg ) \end{aligned}$$

with the weight function defined as

6 $$\begin{aligned} w(t) = \log \frac{p(t)_{\text {nr}}}{p(t)_{\text {er}}}. \end{aligned}$$

The scaling and addition of a factor of 1/2 in Eq. (5) is applied to force the values $L_{\text {recoil}}$ can take to fall between zero and one.

We note that the Gatti parameter [28], which is also commonly used for PSD in scintillation detectors, is formally the same as the $L_{\text {recoil}}$ parameter up to third order in $\frac{p(t)_{\text {nr}}}{p(t)_{\text {er}}}$ [24]. It therefore yields similar PSD performance.

The photon detection time PDFs $p(t)_{\text {nr}}$ and $p(t)_{\text {er}}$ are created by first mixing the singlet and triplet component of the pulse shape with weights from [25] for nuclear recoils and weights as measured in DEAP-3600 for electron recoils. This step is necessary to account for the energy dependence of the singlet fraction. Next, the model PDF is convolved with the detector time resolution from [21] and the TPB fluorescence PDF from [29].^4^ Finally, a flat dark noise component is added. This method can work either with $\text {q}_\text {PE}$ or with $n_{\text {Sc}}$. When using $\text {q}_\text {PE}$, $p(t)_{\text {er}}$ and $p(t)_{\text {nr}}$ are also convolved with the AP PDF.

This method gives us two more PPs:

- $$L_{\text {recoil}}$$ based on $$\text {q}_\text {PE}$$ ($$L^{\text {qpe}}_{\text {recoil}}$$)
- $$L_{\text {recoil}}$$ based on $$n_{\text {Sc}}$$ ($$L^{\text {nsc}}_{\text {recoil}}$$)

## Performance of the 4 PSD parameters

### The DEAP-3600 data in 4 PSD parameters

**Fig. 1:**
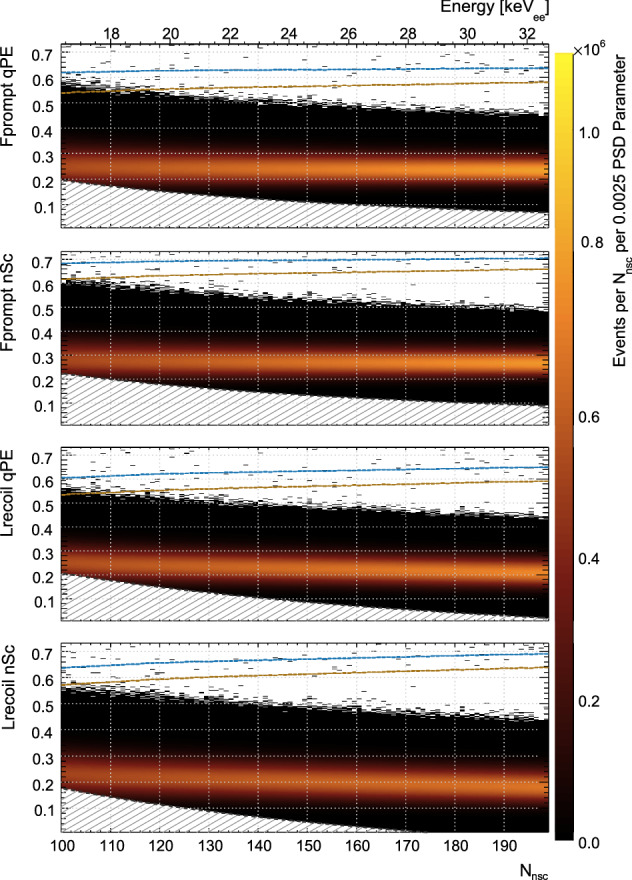
The distributions of mainly $^{39}\text{ Ar }$ $\beta$ decay events for each of the four PSD parameters as a function of energy. The 50% (blue) and 90% (brown) nuclear recoil acceptance lines are also shown. Some events are expected above the $^{39}\text{ Ar }$ population because only a subset of the WIMP analysis cuts are used

Figure 1 shows DEAP-3600 data between approximately 100 $N_{\text {nsc}}$ to 200 $N_{\text {nsc}}$, that is 16 $\hbox {keV}_{\mathrm{ee}}$ to 33 $\hbox {keV}_{\mathrm{ee}}$, binned by $N_{\text {nsc}}$ on the x-axis and the four different PPs discussed in Sect. 4 on the y-axes.

The blue (brown) lines on each sub-figure are the values of the PP above which 50% (90%) of the nuclear recoil signal can be found, based on MC.

In the hatched regions, the trigger efficiency is less than 99.5%. The details of the DEAP-3600 data acquisition system lead to a run- and time-dependent variation in the trigger efficiency, which is calibrated using the method described in [31] and inherently has a large uncertainty. Therefore, rather than applying a correction with an uncertain trigger efficiency value, we do not consider the hatched regions in the analysis.

### The PP distribution model and fits

We want to quantify how close the ER background population is to the NR signal population in Fig. 1 for each PP and as a function of $N_{\text {nsc}}$. We denote the two distributions P$^{\text {ER}}(x, N_{\text {nsc}})$ and P$^{\text {NR}}(x, N_{\text {nsc}})$.

For a background-free WIMP search, a cut in the value of the PP must be chosen such that the probability for a background event to reconstruct as a signal event is $\ll 1$. This requires a discrimination power much better than 1 in $10^{8}$ per keV$_{ee}$. In order to extrapolate the P$^{\text {ER}}(x, N_{\text {nsc}})$ distribution to all possible values of the PP, an empirical model is fit to it. The model describes the 1D PP distribution as a gamma distribution,

7 $$\begin{aligned} \Gamma (x; \mu , b) = \frac{1}{b\mu \Gamma \left( \frac{1}{b}\right) }\left( \frac{x}{b\mu }\right) ^{\frac{1}{b}-1}e^{-\frac{x}{b\mu }} \end{aligned}$$

convolved with a Gaussian:

8 $$\begin{aligned} \Phi (x) = I \cdot \Gamma (x ;\mu , b) *\text {Gauss}(x;\mu =0,\sigma ). \end{aligned}$$

For $L_{\text {recoil}}$, each $N_{\text {nsc}}$-slice is fit individually with this 1D model. The statistical variation between each $N_{\text {nsc}}$-slice leads to leakage predictions that are not perfectly smooth as a function of $N_{\text {nsc}}$, which is inconvenient in the context of constructing a region of interest for a WIMP search. For $F_{\text {prompt}}$, which is the PP used in the WIMP-search analysis, an energy dependence is therefore introduced to $\Phi (x)$ by making the shape parameters and the normalization, $\mu$, *b*, $\sigma$, and *I* functions of $N_{\text {nsc}}$:

9 $$\begin{aligned} b(N_{\text {nsc}})&= a_0 + \frac{a_1}{N_{\text {nsc}}} + \frac{a_2}{N_{\text {nsc}}^2} \end{aligned}$$

10 $$\begin{aligned} \sigma (N_{\text {nsc}})&= a_3 + \frac{a_4}{N_{\text {nsc}}} + \frac{a_5}{N_{\text {nsc}}^2} \end{aligned}$$

11 $$\begin{aligned} \mu (N_{\text {nsc}})&= a_6 + \frac{a_7}{N_{\text {nsc}}} + \frac{a_8}{N_{\text {nsc}}^2} + \frac{a_9}{N_{\text {nsc}}^3} \end{aligned}$$

Thus we obtain a two-dimensional model $\Phi (x,N_{\text {nsc}})$, which results in leakage probabilities that vary smoothly with energy. The functional form of the $N_{\text {nsc}}$-dependence is empirical, and the parameters $a_0,...,a_{9}$ have no physical meaning. The normalization $I(N_{\text {nsc}})$ is not floated in the fit. In the evaluation of $\Phi (x,N_{\text {nsc}})$, $I(N_{\text {nsc}})$ is automatically chosen so that the function value matches the height of the data at the peak of the histogram.

**Fig. 2:**
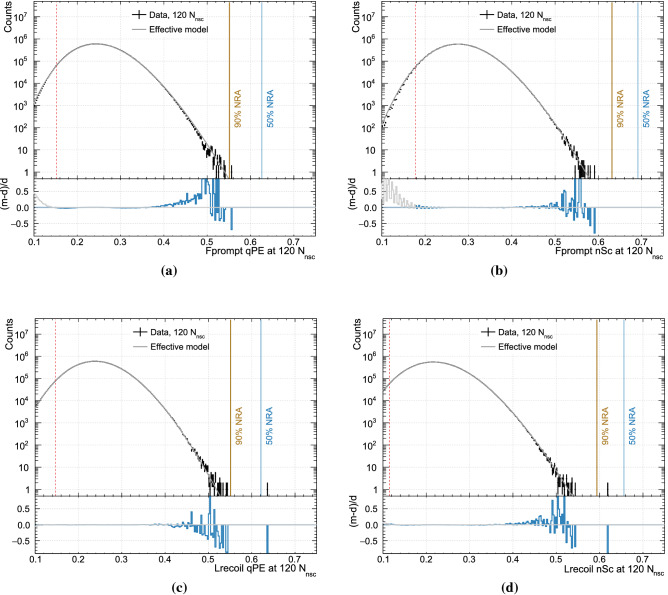
The PP distributions for events at 120 $N_{\text {nsc}}$ (approximately 19.65 $\hbox {keV}_{\mathrm{ee}}$ to 19.82 $\hbox {keV}_{\mathrm{ee}}$) are shown together with the effective model fits. The bin width is 0.0025 and there are $2.8\cdot 10^7$ events in each histogram. The vertical dashed red line marks the $F_{\text {prompt}}$ value above which the trigger efficiency is 99.5%. The brown (blue) line marks a nuclear recoil acceptance of 90% (50%). The lower panel shows the relative deviation between the model and the data

**Fig. 3:**
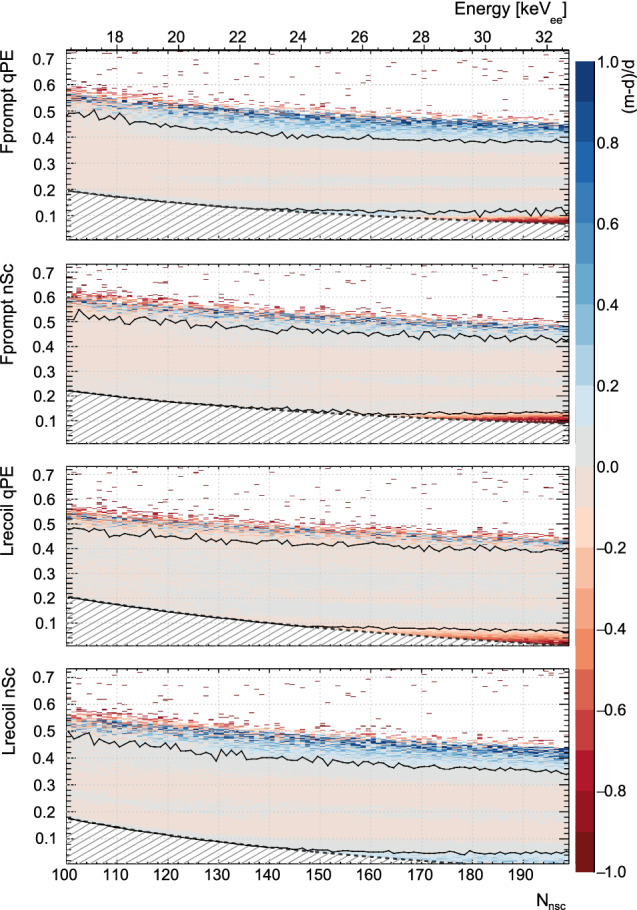
The relative difference, (model-data)/data, for each of the four PSD parameters. The only significant deviations between model and data are at the edge of the distributions, where several systematic effects bias the event count (see Sect. 5.4). The black contour shows, for each $N_{\text {nsc}}$ slice, the first bin when going from the center of the population to higher/lower values of the PP where the absolute value of the difference between model and data is more than 10%

Figure 2 shows the fit results at 120 $N_{\text {nsc}}$(this includes events with a value of $N_{\text {nsc}}$ in [120, 121)). Figure 3 shows the relative difference between model and data over the 2D space for each PP. The fit is performed over a range on the energy axis of [98:210] $N_{\text {nsc}}$, and on the PP axis from the upper edge of the trigger-efficiency curve to 0.6.

### Leakage predictions

We judge the performance of a PP by the probability of a background event to leak into the signal region as a function of $N_{\text {nsc}}$ and nuclear recoil acceptance (NRA). Figure 1 shows the means of the background and signal distributions becoming closer toward lower energies, which is expected from the energy-dependence of the singlet to triplet ratio. The separation between the means of the populations is therefore a function of energy. The spread of the distributions about the mean is largely determined by counting statistics, that is by $N_{\text {nsc}}$. Hence, it is natural to consider the leakage probability in bins that are one $N_{\text {nsc}}$ wide.

The NRA is the fraction of signal events in the range [*x*, 1],

12 $$\begin{aligned} \text {NRA}(x, N_{\text {nsc}}) = \frac{\int _{x}^1 P^{\text {NR}}(x', N_{\text {nsc}}) dx'}{\int _{0}^1 P^{\text {NR}}(x', N_{\text {nsc}}) dx'} \; . \end{aligned}$$

The uncertainty on the NRA is calculated using the binomial confidence interval (the Wilson score) at 95% coverage.

The fraction of ER background events that leaks into the region [*x*, 1] is defined similarly as

13 $$\begin{aligned} P_{\text {leak}}(x,N_{\text {nsc}}) = \frac{ \int _x^1 \Phi (x', N_{\text {nsc}})dx' }{\int _0^1 \Phi (x', N_{\text {nsc}})dx' } \end{aligned}$$

where we use the mathematical model $\Phi (x,N_{\text {nsc}})$ rather than the data $P^{\text {ER}}(x, N_{\text {nsc}})$.

The uncertainty on $P_{\text {leak}}(x,N_{\text {nsc}})$ is determined by assuming the parameter uncertainties returned by the fitter are Gaussian and randomly drawing parameter combinations from a multidimensional Gaussian with mean and standard deviation for each parameter and correlations between parameters taken from the fit result. For each parameter combination, $P_{\text {leak}}^i(x,N_{\text {nsc}})$ is calculated, where *i* stands for the *i*^th^ set of randomly drawn parameters. For each x, the central value of the leakage probability is the mean over all $P_{\text {leak}}^i(x,N_{\text {nsc}})$ (this reproduces the curve obtained from the parameter values returned by the fit), and the up (down) uncertainties are the standard deviations among all cases where $P_{\text {leak}}^i(x,N_{\text {nsc}})$ is bigger (smaller) than the nominal value.

**Fig. 4:**
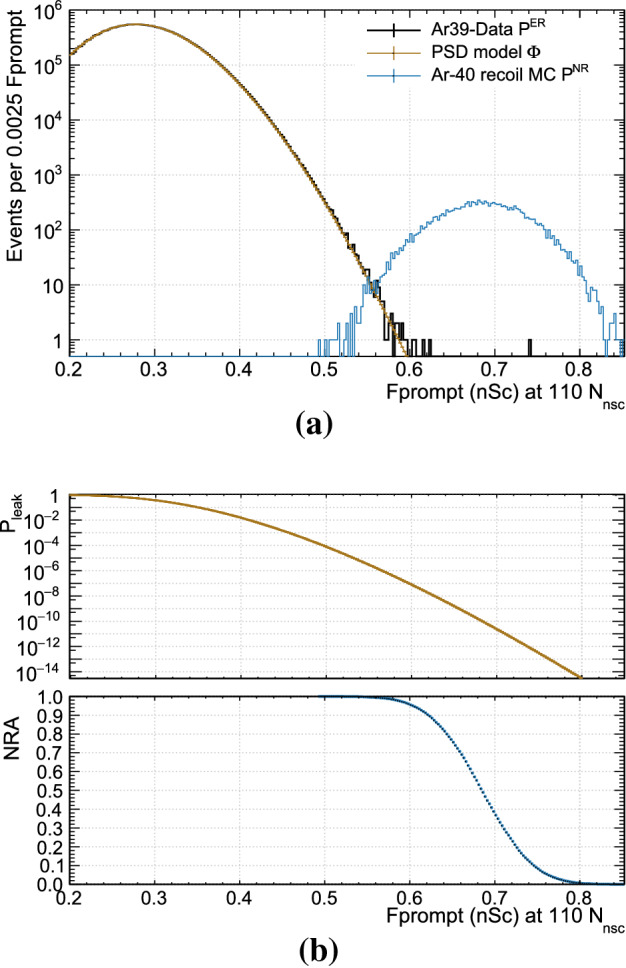
**a** The $F^{\text {nsc}}_{\text {prompt}}$ distributions at 110 $N_{\text {nsc}}$ are shown for $^{39}\text{ Ar }$ $\beta$ events (background), together with the model fit, and for simulated $^{40}\text{ Ar }$ recoil events (signal). **b** The background leakage probability (based on the fit model to $^{39}\text{ Ar }$ data) and signal acceptance (based on signal MC) as a function of the PSD parameter is shown

Figure 4a shows $P^{\text {ER}}$, $\Phi$, and $P^{\text {NR}}$, and (b) shows the respective NRA and $P_{\text {leak}}$, all for $F^{\text {nsc}}_{\text {prompt}}$ and at $N_{\text {nsc}}$=110.

**Fig. 5:**
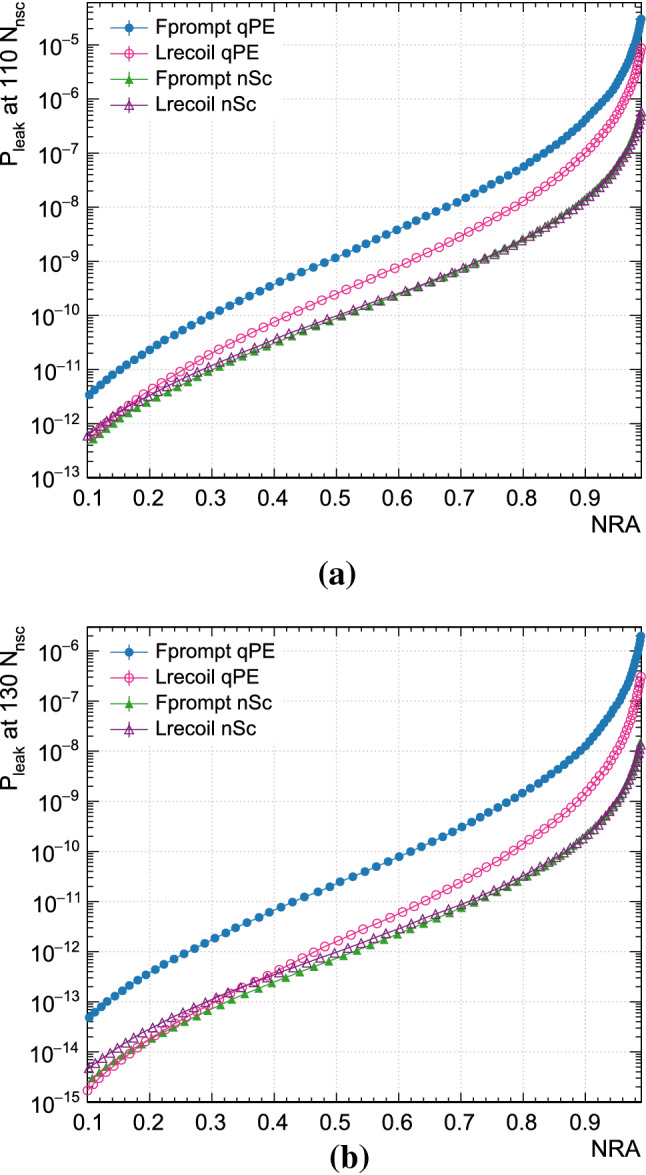
Leakage probabilities for each PP as a function of NRA for events at **a** 110 $N_{\text {nsc}}$(approximately 17.46 $\hbox {keV}_{\mathrm{ee}}$ to 17.61 $\hbox {keV}_{\mathrm{ee}}$) and at **b** 130 $N_{\text {nsc}}$(approximately 19.65 $\hbox {keV}_{\mathrm{ee}}$ to 19.82 $\hbox {keV}_{\mathrm{ee}}$). Statistical error bars, where not visible, are smaller than the marker size

In Fig. 5, the leakage probability is shown as a function of NRA for all four PPs for $N_{\text {nsc}}$=110 and 130. We chose to focus on 110 $N_{\text {nsc}}$ because this is close to the lower threshold relevant to the DEAP-3600 detector, and on 130 $N_{\text {nsc}}$ because this is where the leakage probability even at 90% NRA reaches approximately $1\times 10^{-10}$, two orders of magnitude better than required. At higher $N_{\text {nsc}}$, the separation between background and signal populations is thus so good that PSD leakage is no longer as relevant.

**Fig. 6:**
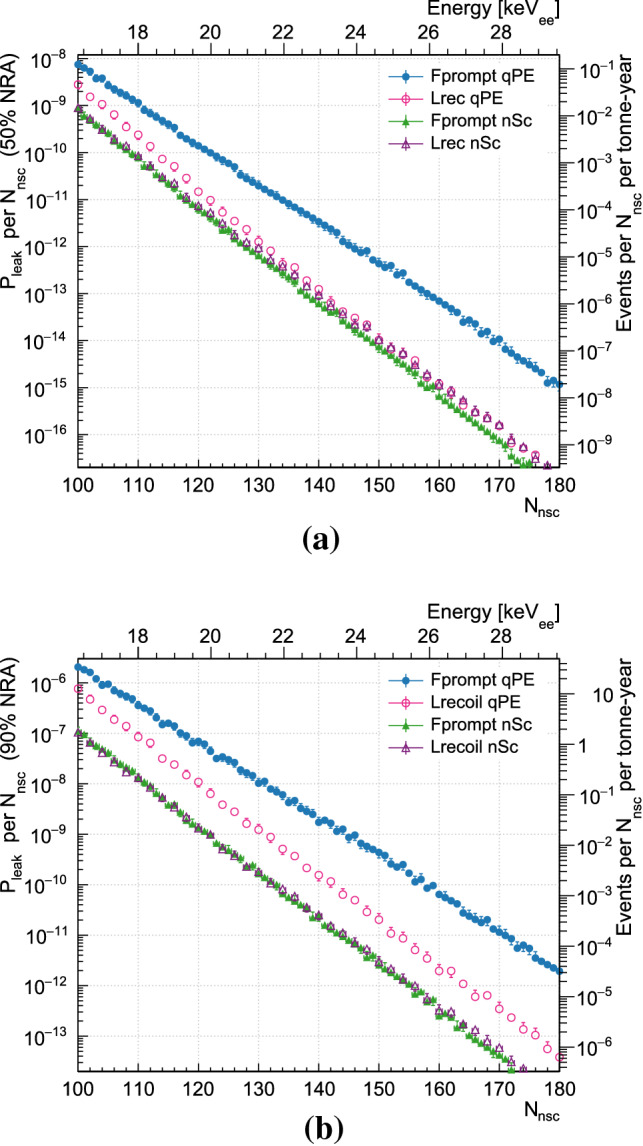
The leakage probability as a function of $N_{\text {nsc}}$ at **a** 50% and **b** 90% NRA. Statistical error bars, where not visible, are smaller than the marker size

For 50% and 90% NRA, Fig. 6 shows as a function of energy the leakage probability and the number of leaked events per $N_{\text {nsc}}$ this would correspond to in a nominal 1 tonne year exposure. The error bars are dominated by the uncertainty in the NRA position and are correlated between bins. The statistical uncertainty from the fit is negligible.

### Systematic uncertainties

#### Pile-up

The data contain pile-up events from (a) random coincidences between two $^{39}\text{ Ar }$ decays (b) random coincidences of $^{39}\text{ Ar }$ with Cherenkov light produced in the acrylic, and (c) correlated pile-up between Cherenkov light in the acrylic or PMT glass and an ER event (other than $^{39}\text{ Ar }$ beta decay) in the LAr, for example from a gamma emitted by $^{208}\text{ Tl }$ in the PMT glass that interacts both in the acrylic and in the LAr. While LAr scintillation timing has a double-exponential decay time structure, Cherenkov light is emitted in a flash shorter than the time resolution of the detector.

The pile-up cuts described in Sect. 3.1 were designed specifically to remove these events [20]. Based on MC, Table 1 gives an estimate of the fraction of the data that still contains pile-up after pile-up cuts. Fractions are given separately for the second event occurring within the prompt and the late $F_{\text {prompt}}$ window. Coincidences within the prompt window tend to make the event more nuclear-recoil-like, while those in the late window tend to make the event look less like a nuclear recoil.

$^{39}\text{ Ar }$ events that have either another $^{39}\text{ Ar }$ event or Cherenkov light randomly piled-up in the late time window are the most common coincidences. Their effect is small though, because the second event can have at most 10% as much light as the first event for the pile-up cuts not to remove it. For example, a 10 PE event ($^{39}\text{ Ar }$ or Cherenkov) piling up in the late part of a 120 PE $^{39}\text{ Ar }$ event with $F_{\text {prompt}}$=0.3 creates a pile-up event reconstructing at 130 PE and $F_{\text {prompt}}$=0.277. We verified through a toy MC that this level of contamination with pile-up in the late window does not affect the leakage predictions.

The next most common type of pile-up is correlated gamma-Cherenkov events. For example, 10 PE from Cherenkov light in prompt coincidence with a 120 PE ER event with $F_{\text {prompt}}$=0.3 create a pile-up event reconstructing at 130 PE and $F_{\text {prompt}}$=0.35. We simulated Cherenkov light production in PMT glass and acrylic as well as energy deposition in the LAr from $^{208}\text{ Tl }$ in the PMT glass using the full detector MC to obtain the event rate and PP distributions in the energy region of 100 $N_{\text {nsc}}$ to 200 $N_{\text {nsc}}$. We then verified through a toy MC that the admixture of these events did not affect the leakage predictions for pure ER events, which this paper is concerned with. Based on the MC, the leakage of correlated pile-up events starts to dominate over that of pure ER events at a leakage probability for pure events smaller than $10^{-13}$.

#### Non-ER events, blinding, and energy-dependence of the shape parameters

The cuts used do not remove all alpha events with degraded energy. These events reconstruct just above the $^{39}\text{ Ar }$-population in the PP. This leads to a small increase in the number of events at the upper edge of the $^{39}\text{ Ar }$ population. At the same time, data blinding removes some events from this region.

The data blinding is implemented in low-level variables that do not correspond exactly to the PPs and energy estimator used here, hence there is no sharp line above which blinding affects the data in the variables shown here. The blinding removes fewer than 10 events per $N_{\text {nsc}}$ from the upper edge of the PP distributions.

**Fig. 7:**
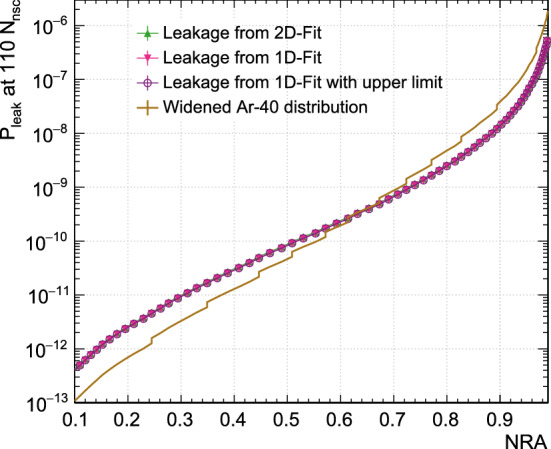
A study of three effects that lead to systematic uncertainties on the leakage probabilities. The green up-triangles show the nominal leakage probability for $F^{\text {nsc}}_{\text {prompt}}$ from the 2D fit (the curve is the same as in Fig. 5). The pink down-triangles use the results from the 1D fit instead. The purple boxes are obtained with the 1D fit where the upper fit limit was reduced from 0.6 to 0.55 to exclude bins with fewer than 10 events. The points overlap, indicating that the fit procedure does not significantly change the leakage predictions. The brown curve uses an $^{40}\text{ Ar }$ distribution modified to account for energy resolution differences between MC and real data [10]

To study the effect that events at the upper edge of the $^{39}\text{ Ar }$ PP distribution have on the fit, all fits were repeated with an upper fit limit in the PP parameter set to be approximately the last bin that still has more than 10 events. Furthermore, these fits were done using the 1D model, Eq. (8), to additionally test the difference between the two modelling approaches. Figure 7 shows leakage predictions for $F^{\text {nsc}}_{\text {prompt}}$ based on the standard fit with the 2D model, based on the fit with the full fit range in $F^{\text {nsc}}_{\text {prompt}}$ using the 1D model, and based on the fit with restricted fit range in $F^{\text {nsc}}_{\text {prompt}}$ using the 1D model.

**Table 1:** Estimated fractions of events between 100 $N_{\text {nsc}}$ to 200 $N_{\text {nsc}}$ that are piled-up with a second event and not removed by the cuts, for different types of coincidences. ‘Timing’ distinguishes between coincidences that occur within the prompt $F_{\text {prompt}}$ window and those that occur within the late $F_{\text {prompt}}$ window

Partners	Timing	Fraction
Random coincidences		
$^{39}\text{ Ar }$-$^{39}\text{ Ar }$	Prompt	4$\cdot 10^{-6}$
$^{39}\text{ Ar }$-$^{39}\text{ Ar }$	Late	3$\cdot 10^{-4}$
$^{39}\text{ Ar }$-Cherenkov	Prompt	$\le 4\cdot 10^{-7}$
$^{39}\text{ Ar }$-Cherenkov	Late	$\le$ 1$\cdot 10^{-4}$
Correlated events		
Gamma-Cherenkov	Prompt	$\le 6\cdot 10^{-5}$

#### Modelling of the NR signal region

The position of the dark-matter signal region in each PP has systematic uncertainties related to how well the MC describes the detector and to incomplete knowledge of the ratio of singlet to triplet states created as a function of energy for nuclear recoil events. We consider here only the possible mis-modelling of the detector energy resolution, which has a significant effect on the width of the $^{40}\text{ Ar }$ distribution and a slight effect on its mean. For the $F_{\text {prompt}}$ parameters, we construct alternate $^{40}\text{ Ar }$ event distributions with a larger energy resolution consistent with the width of the $^{39}\text{ Ar }$ PP distribution, as described in [10]. The leakage predictions based on this modified signal region are shown in Fig. 7.

#### Data cleaning cuts

After applying low-level and timing cuts, and the pile-up cut that requires that there is no more than one event peak within the digitization window, varying the cut values for the remaining cuts in reasonable ranges does not change the shape of the PP distribution in any significant way.

## Stability of the discrimination power in time

**Fig. 8:**
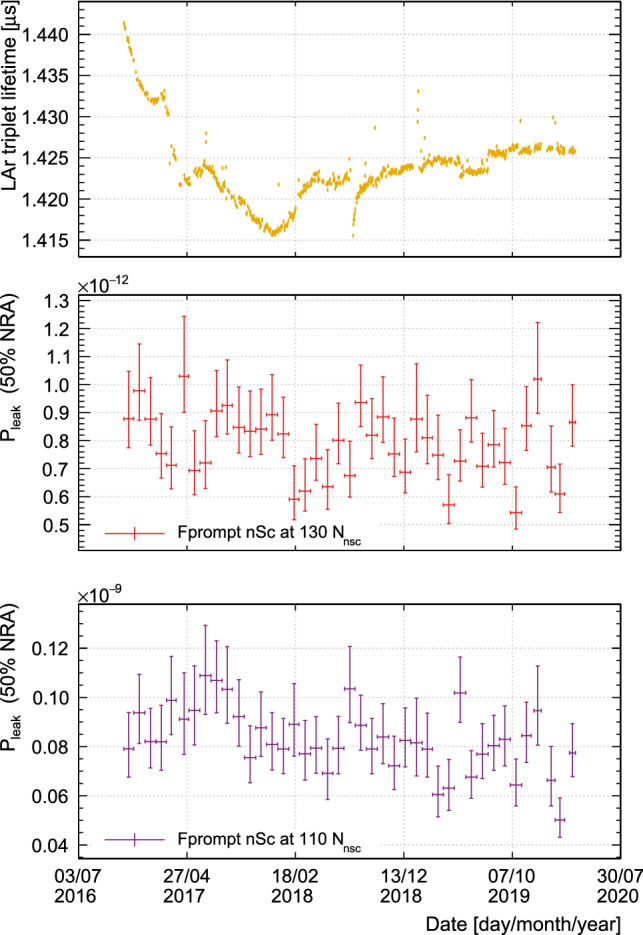
The upper two figures show the time evolution of the leakage probabilities at 110 $N_{\text {nsc}}$ and 130 $N_{\text {nsc}}$ for $F^{\text {nsc}}_{\text {prompt}}$ at 50% NRA. The triplet lifetimes in the third figure are determined by using the full mathematical model of the $^{39}\text{ Ar }$ scintillation pulse shape [22]. The observed triplet lifetime strongly depends on the state variables of the detector, e.g. temperature, pressure, or impurities diffused from the detector into the liquid argon. A change in the triplet lifetime is expected to influence the shape of the PP distribution and hence causes the PP distribution of the whole data set to be a superposition of distributions with slightly different parameters

The data shown here cover approximately 3.5 years of real time. We determined the leakage predictions individually per month to check if there were any changes due to detector degradation. Calibrations show no significant degradation of the light detection system, so the most significant effects are expected from changes in light yield or in the observed LAr triplet excimer lifetime due to impurities in the LAr. The triplet lifetime, which is sensitive to electro-negative impurities [32, 33], is shown in Fig. 8 for each day when physics data were recorded. The triplet lifetime is obtained from fits to the $^{39}\text{ Ar }$-pulse shape of events in the energy region considered here [22]. The change over 3.5 years is less than 2%. For the $F_{\text {prompt}}$ PPs, this corresponds to a shift in the position of the PP distribution mean for the $^{39}\text{ Ar }$ ($^{40}\text{ Ar }$) population of approximately 0.8% (0.4%).

The leakage predictions shown in Fig. 8 are calculated against a fixed nuclear recoil band, that is the position of the NRA lines was not adjusted for changes in triplet lifetime, since the change in leakage predictions due to the small shift in the $^{40}\text{ Ar }$’s PP-distribution mean is of approximately the same magnitude as the statistical uncertainty from the fit. We see no significant degradation in PSD performance over the operation time of the DEAP-3600 detector.

## Discussion

We consider the PSD parameter distribution for approximately $2.9\cdot 10^9$ events from 16 $\hbox {keV}_{\mathrm{ee}}$ to 33 $\hbox {keV}_{\mathrm{ee}}$, most of which represent electromagnetic interactions, largely $^{39}\text{ Ar }$ $\beta$-decays. The data contain a small fraction of pile-up events that do not significantly alter the distribution shape. Figure 3 shows that the effective fit model for the PP-distribution shape of ER events describes the data to better than 10% accuracy over several orders of magnitude. Starting near the upper edge of the population of electromagnetic events, the model no longer describes the data well. In this region, non-ER backgrounds appear, and the event count is further biased by data blinding. This region does not significantly influence the model fit, as shown in Fig. 7.

Based on the model fit, we calculate the probability for ER backgrounds to leak into the NR signal region. The latter is determined using Monte Carlo simulation. The simulation does not perfectly describe the shape of the PP distributions. For the $F^{\text {nsc}}_{\text {prompt}}$ PP, an NR distribution shape is constructed to match measured shapes. The leakage probabilities shown in the remaining figures are systematically high or low by an amount as shown in Fig. 7, but the relative difference between the performance of the PPs remains the same.

Figure 5 shows that $L^{\text {qpe}}_{\text {recoil}}$ is an improvement over $F^{\text {qpe}}_{\text {prompt}}$, but $L^{\text {nsc}}_{\text {recoil}}$ provides little to no improvement over $F^{\text {nsc}}_{\text {prompt}}$. The fact that the $L_{\text {recoil}}$ algorithm barely improves on $F_{\text {prompt}}$ if the input is not significantly biased, even though $L_{\text {recoil}}$ uses the full information available about photon arrival times, while $F_{\text {prompt}}$ only considers whether or not photons arrived in the prompt window, can be understood by considering the weight functions.

The weight functions for the likelihood-based PP (Eq. 6) can be seen as the information carried by a photon as a function of the photon arrival time. A weight of $+1$ means the photon points toward the NR-hypothesis, while a weight of $-1$ means the photon points toward the ER-hypothesis. A weight of 0 means that a photon detected at this time carries no information about the interaction type.

To compare the photon weight functions for $F_{\text {prompt}}$ and $L_{\text {recoil}}$, the weight function for $F_{\text {prompt}}$ is manually constructed as a step function: It is 1 in the prompt window (all photons here count toward the NR hypothesis), then $-1$ until the end of the total integration time (all photons here count toward the ER hypothesis), and 0 at all later times (since they are not considered).

**Fig. 9:**
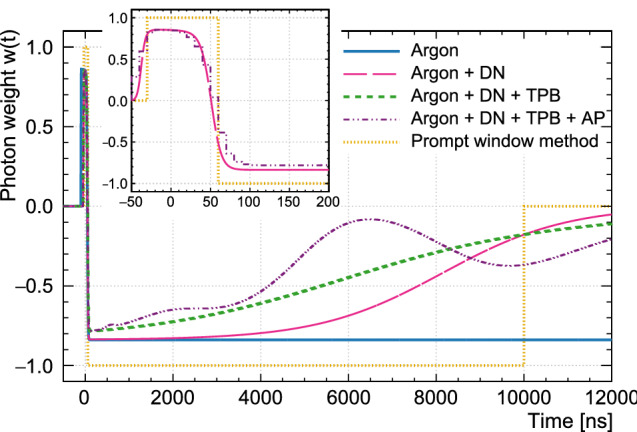
The $L_{\text {recoil}}$ weight-functions from Eq. (6) calculated for photon detection PDFs that take into account different components of the detector response are shown. The blue solid line considers only the LAr scintillation (Argon), the pink long-dashed line adds dark noise (DN), the green short-dash line includes the slow component of TPB fluorescence (TPB), and the purple dot-dash line adds afterpulsing (AP). This is compared to the equivalent weight function used in the $F_{\text {prompt}}$ PP (yellow dotted line). The insert zooms in on the time region from $-$ 50 to 200 ns; for reasons of legibility, only three of the lines are shown here. Note that the detector’s time resolution is included in the PDFs the photon weights are based on. The weight can be interpreted as follows: PE detected at times where $w(t) > 0$ strengthen the NR hypothesis, while PE detected where $w(t) < 0$ strengthen the ER hypothesis

The weight functions for $F_{\text {prompt}}$ and for $L_{\text {recoil}}$ are shown together in Fig. 9. The $L_{\text {recoil}}$ weight function is shown for PDFs that include different detector effects, to illustrate their impact. The weights are always close to $+1$ near $t=0$, where most of the singlet light is detected. In the absence of detector effects, the weight function falls sharply after the singlet peak and stays constant afterwards, because at later times, the singlet component becomes negligible and the photon weight becomes the log-ratio of triplet fractions for ER and NR events. This shape is very similar to the step-function that is used for $F_{\text {prompt}}$. This means that the $F_{\text {prompt}}$ parameter already captures nearly all the available information.

When adding delayed TPB fluorescence and dark noise to the PDFs, the weight function still drops sharply after the singlet peak but then slowly rises towards zero as the signal-to-noise ratio becomes worse. Including AP in the model adds bumps to the weight function at the times of high AP probability; due to the AP, photons at this time contribute little information, which $L^{\text {qpe}}_{\text {recoil}}$ accounts for but $F^{\text {qpe}}_{\text {prompt}}$ does not.

$L^{\text {qpe}}_{\text {recoil}}$ cannot reliably reach the performance of $L^{\text {nsc}}_{\text {recoil}}$ because $L^{\text {qpe}}_{\text {recoil}}$ accounts for AP based on the detected photon times over all PMTs. The PE counting algorithm used to determine $n_{\text {Sc}}$ is applied individually for each PMT, where an AP must follow a previous pulse in the same PMT, so it uses more of the available information to make a better assessment as to whether or not a pulse is an AP.

## Conclusion

We presented how approximately $2.9\cdot 10^9$ $^{39}\text{ Ar }$ beta decay events between approximately 16 $\hbox {keV}_{\mathrm{ee}}$ to 33 $\hbox {keV}_{\mathrm{ee}}$, collected by the DEAP-3600 detector in 840 live-days, look under four different pulse-shape discrimination methods, and predict their leakage probabilities into the nuclear-recoil signal region as a function of energy and nuclear recoil acceptance. With a light yield of 6.05 $n_{\text {Sc}}$/keV$_{ee}$, in a 0.165 $\hbox {keV}_{\mathrm{ee}}$ wide bin at 16 keV and allowing for a 50% NRA, we estimate that with the $F^{\text {qpe}}_{\text {prompt}}$ PSD parameter a leakage of $7.5\cdot 10^{-9}$ is achieved, while $L^{\text {qpe}}_{\text {recoil}}$ reaches $2.3\cdot 10^{-9}$, and $F^{\text {nsc}}_{\text {prompt}}$ and $L^{\text {nsc}}_{\text {recoil}}$ both reach approximately $1\cdot 10^{-9}$.

We find that due to the time structure of LAr scintillation, likelihood-based methods only improve on the prompt-fraction method if there is significant instrumental bias, in this case PMT afterpulsing, and information on this bias is included in the likelihood analysis. Otherwise, the prompt-fraction method captures nearly all the available information, leaving little room for the likelihood analysis to make a better assessment as to the particle type.

## Data Availability

This manuscript has no associated data or the data will not be deposited. [Authors’ comment: The data used in this paper requires approximately 600TB of storage space, and its interpretation requires extensive understanding of the detector. Therefore, making the data publicly available is impractical. Access to the data can be granted on request to the DEAP collaboration.]

## Footnotes

1 The term refers to events from particles that cause ionization and excitation mainly by interacting with the electrons in the argon atom, e.g. electrons, photons, or muons.

2 The widths of the ER distribution $\sigma _{er}$ and the NR distribution $\sigma _{nr}$ are approximately the same, therefore $\sigma _{nr}$ is not explicitly included in the calculation.

3 This is assuming the time resolution is good enough that all of the singlet light is detected inside the prompt time window.

4 For the shape of the slow TPB fluorescence, [29] is superseded by [30], but both shapes are very similar at the time scales of interest here.
